# Impact of Chaos Functions on Modern Swarm Optimizers

**DOI:** 10.1371/journal.pone.0158738

**Published:** 2016-07-13

**Authors:** E. Emary, Hossam M. Zawbaa

**Affiliations:** 1 Department of Information Technology, Faculty of Computers and Information, Cairo University, Cairo, Egypt; 2 Computing Department, Faculty of Computer Studies, Arab Open University, Shrouq, Egypt; 3 Department of Information Technology, Faculty of Computers and Information, Beni-Suef University, Beni-Suef, Egypt; 4 Department of Computer Science, Faculty of Mathematics and Computer Science, Babes-Bolyai University, Cluj-Napoca, Romania; Jiangnan University, CHINA

## Abstract

Exploration and exploitation are two essential components for any optimization algorithm. Much exploration leads to oscillation and premature convergence while too much exploitation slows down the optimization algorithm and the optimizer may be stuck in local minima. Therefore, balancing the rates of exploration and exploitation at the optimization lifetime is a challenge. This study evaluates the impact of using chaos-based control of exploration/exploitation rates against using the systematic native control. Three modern algorithms were used in the study namely grey wolf optimizer (GWO), antlion optimizer (ALO) and moth-flame optimizer (MFO) in the domain of machine learning for feature selection. Results on a set of standard machine learning data using a set of assessment indicators prove advance in optimization algorithm performance when using variational repeated periods of declined exploration rates over using systematically decreased exploration rates.

## 1 Introduction

Chaos is the form of non-linear movement of the dynamics system and can experience any state according to its own regularity. Chaos has the ability of information processing that is applied widely in many fields like optimization problems, machine learning, and pattern recognition. The searching techniques based on chaos are similar to genetic algorithms (GA), but it uses the chaos variables. Because of the chaos is not repetition system, it can execute the overall search process in less computational time than stochastic ergodic techniques based on the probability [1]. Lately, the thought of applying the chaotic systems rather than random operations has been observed in the optimization theory. The randomness role can be worked by chaotic motion rather than random processes in the optimization algorithms [2]. Numerous studies state as true that the advantages of applying the chaotic-based processes instead of random-based processes are usually visible despite the fact that a general principle can not be planned [3]. Chaos optimization algorithm (COA) has the chaos properties like ergodicity that can more efficiently escape from local minima than whatever other stochastic optimization algorithm [2]. COA utilizes the consistency exist as a part of a chaotic motion to escape from local minima, but the random-based procedures escape from the local minima by perceiving some unacceptable solutions with a particular probability [2, 4, 5].

Over the previous years, the number of features in machine learning and pattern recognition applications have expanded from hundreds to thousands of input features. Feature selection is a procedure for distinguishing the subset of significance features and remove the repetitive ones. Additionally, this technique is valuable when the size of input features is vast and not every one of them are required for describing the data [6]. Feature selection prompts the decrease of the dimensionality of features space that will enhance the classification performance. Moreover, it will help in better data understanding and reduce the computation time during the classification process [7, 8]. There are two different techniques of feature selection approaches: filter-based and wrapper-based [9]. The *filter-based approach* searches for a subset of features that optimize a given data-dependent criterion [10]. The *wrapper-based approach* applies the machine learning strategy as the evaluation component that has better results than the filter-based approach [11]; however, it is computationally costly [12].

Generally, feature selection is formulated as a *multi-objective* problem with two goals: *minimize* the number of selected features and *maximize* the classification performance. These two goals are conflicting and the optimal solution ought to adjust between them. A sort of search methods has been applied, for example, sequential forward selection (SFS) [13] and sequential backward selection (SBS) [14]. Nonetheless, these feature selection methods still experience the ill effects of stagnation in the local optima [15]. Evolutionary computing (EC) algorithms adaptively search the space by using a set of search operators that contact in a social way to achieve the optimal solution [16, 17].

The chaos genetic feature selection optimization method (CGFSO) investigates the search space with every conceivable combination of a given data set. Each individual in the population represents a candidate solution and the feature dimension is the same length as chromosomes length [18, 19]. The chaotic time series with an EPNet algorithm requires a small network architecture whereas the expansion of neural parts might debase the performance amid the evolution and gives more survival probabilities to smaller networks in the population [20, 21]. The chaotic antlion optimization (CALO) can converge to the same optimal solution for a higher number of applications regardless of the stochastic searching and the chaotic adjustment [22].

In this paper, our aim is to propose an optimization algorithm based on two chaotic functions that employed to analyze their performance and impact on three bio-inspired optimization algorithms namely grey wolf optimization (GWO), antlion optimization (ALO), and moth-flame optimization (MFO). The rest of this paper is comprised as follows: Section 2 gives the foundation information of three used bio-inspired optimization algorithms and describes the usage of chaotic variants of GWO, ALO, and MFO in feature selection. Experimental results with discussions are displayed in Section 3 and the conclusions and future work are provided in Section 4.

## 2 Materials and Methods

The following subsections provide the short brief of the applied three bio-inspired optimization algorithms and chaotic maps. After that, it describes the usage of chaotic variants of GWO, ALO, and MFO in feature selection.

### 2.1 Preliminaries

This subsection provides a brief summary of the three modern bio-inspired optimizers namely grey wolf optimization (GWO), antlion optimization (ALO), and moth-flame optimization (MFO). Moreover, we have provided a short introduction to the chaos theory.

1. **Gray wolf optimization (GWO)**
  GWO is a relatively new optimization algorithm proposed by Mirjalili et al. in 2014 [23]. This algorithm mimics the hunting process of grey wolves. Grey wolves often live in a pack with strict leadership hierarchy. Table 1 describes such hierarchy and the responsibility of each agent in the pack.
  For a pack to hunt prey, a set of phases are to be applied in sequence [24]:
  **Prey encircling**: the pack encircles a prey by repositioning individual agents according to the prey location. The encircling is modeled as in given Eq (1):
  $$\begin{array}{c} \vec{X}\left(t+1\right)={\vec{X}}_{p}\left(t\right)+\vec{A}.\vec{D}, \end{array}$$ (1)
  where $\vec{D}$ is as defined in Eq (2), *t* is the iteration number, $\vec{A}$ and $\vec{C}$ are coefficient vectors, ${\vec{X}}_{p}$ is the prey position, and $\vec{X}$ is the gray wolf position.
  $$\begin{array}{c} \vec{D}=\left|\vec{C}.{\vec{X}}_{p}\left(t\right)-\vec{X}\left(t\right)\right|, \end{array}$$ (2)
  where $\vec{A}$, $\vec{C}$ vectors are calculated as in Eqs (3) and (4).
  $$\begin{array}{c} \vec{A}=2a.\vec{r_{1}}-a, \end{array}$$ (3)
  $$\begin{array}{c} \vec{C}=2\vec{r_{2}}, \end{array}$$ (4)
  where *a* is linearly decreased from 2 to 0 over the course of iterations controlling *exploration* and *exploitation*, and *r*_1_, *r*_2_ are random vectors in [0, 1]. The value of *a* is the same for all wolves in the pack.
  By the end of encircling stage, the hunting stages start.
  **Hunting**: is performed by the whole pack based on the information coming from the alpha, beta and delta wolves which are expected to know the prey location. Alpha, beta, and delta are assumed to have better knowledge about the potential location of prey and hence are used as guides for hunting. Individual wolf in the pack updates its position as shown in the Eq (5):
  $$\begin{array}{c} \vec{X}\left(t+1\right)=\frac{\vec{X_{1}}+\vec{X_{2}}+\vec{X_{3}}}{3}, \end{array}$$ (5)
  where $\vec{X_{1}}$, $\vec{X_{2}}$, $\vec{X_{3}}$ are defined as in Eqs (6), (7) and (8) respectively.
  $$\begin{array}{c} \vec{X_{1}}=\left|\vec{X_{\alpha }}-\vec{A_{1}}.\vec{D_{\alpha }}\right|, \end{array}$$ (6)
  $$\begin{array}{c} \vec{X_{2}}=\left|\vec{X_{\beta }}-\vec{A_{2}}.\vec{D_{\beta }}\right|, \end{array}$$ (7)
  $$\begin{array}{c} \vec{X_{3}}=\left|\vec{X_{\delta }}-\vec{A_{3}}.\vec{D_{\delta }}\right|, \end{array}$$ (8)
  where $\vec{X_{\alpha }}$, $\vec{X_{\beta }}$, $\vec{X_{\delta }}$ are the first three best solutions in the swarm at a given iteration *t*, $\vec{A_{1}}$, $\vec{A_{2}}$, $\vec{A_{3}}$ are defined as in Eq (3), and $\vec{D_{\alpha }}$, $\vec{D_{\beta }}$, $\vec{D_{\gamma }}$ are defined using Eqs (9), (10) and (11) respectively.
  $$\begin{array}{c} \vec{D_{\alpha }}=\left|\vec{C_{1}}.\vec{X_{\alpha }}-\vec{X}\right|, \end{array}$$ (9)
  $$\begin{array}{c} \vec{D_{\beta }}=\left|\vec{C_{2}}.\vec{X_{\beta }}-\vec{X}\right|, \end{array}$$ (10)
  $$\begin{array}{c} \vec{D_{\delta }}=\left|\vec{C_{3}}.\vec{X_{\delta }}-\vec{X}\right|, \end{array}$$ (11)
  where $\vec{C_{1}}$, $\vec{C_{2}}$, and $\vec{C_{3}}$ are defined as in Eq (4).
  By the end of preying stage, the pack attacks the prey in what is called attacking stage.
  **Attacking stage**: the attacking phase is performed by allowing the agents to approach the prey which is achieved by decrementing the exploration rate *a* as outlined in Eq 4 with *a* ∈ [1, 2].
  By finishing the attacking phase the pack is ready to search for a new prey which is the next phase.
  **Search for prey**: in this phase, wolves diverge from each other to find a new prey. This behavior is modeled by allowing large value for the parameter *a* to allow for exploration of the search space as outlined in Eq 4 with *a* ∈ [0, 1].
  Algorithm 1 outlines the gray wolf optimization (GWO) algorithm.
  **Algorithm 1:** Grey wolf optimization (GWO) algorithm
  1: **Inputs**: *n* Number of gray wolves in the pack,
    *N*_*Iter*_ Number of iterations for optimization.
  2: **Outputs**: *x*_*α*_ Optimal gray wolf position,
    *f*(*x*_*α*_) Best fitness value.
  3: Initialize a population of *n* gray wolves positions randomly.
  4: Find the *α*, *β*, and *δ* solutions based on their fitness values.
  5: **while** stopping criteria is not met **do**
  6:   **for all** *Wolf*_*i*_ ∈ *pack* **do**
  7:    Update current wolf’s position according to Eq (5).
  8:   **end for**
  9:   Update *a*, *A*, and *C* as in Eqs (2) and (3).
  10:  Evaluate the positions of individual wolves.
  11:  Update *α*, *β*, and *δ* positions as in Eqs (9), (10) and (11).
  12: **end while**
  13: Select the optimal position (*x*_*α*_) and its fitness (*f*(*x*_*α*_)).
2. **Antlion optimization (ALO**)
  ALO is a recent optimization algorithm that was proposed by Mirjalili in 2015 [25]. ALO algorithm emulates the chasing system of the antlions in nature. There are two different agents utilized as a part of ALO algorithm to be specific *ants* and *antlions*. Antlion always dependably burrows a trap to chase ants, and when an ant falls in the hole, the antlion tosses sand towards it until it is slaughtered and ingested. After that, the antlion readies the trap again for the next chase.
  In the artificial optimizer, the ants change their positions according to the antlions positions. Individual ant performs two local searches around the elite antlion and the selected antlion; then updates itself position as the average of these two random walks as in Eq (12):
  $$\begin{array}{c} Ant_{i}^{t}=\frac{R_{A}^{t}+R_{E}^{t}}{2}, \end{array}$$ (12)
  where $R_{A}^{t}$ is the random walk around the roulette wheel selected antlion, and $R_{E}^{t}$ is the random walk around the elite antlion.
  The random walking of an ant $X_{ant_{i}}^{t}$ around a given antlion $X_{antlion_{j}}^{t}$ is modeled as in the Eq (13):
  $$\begin{array}{c} X_{ant_{i}}^{t}=\frac{\left(X_{i}-a_{i}\right)\times \left(d_{i}-c_{i}^{t}\right)}{\left(b_{i}^{t}-a_{i}\right)}+c_{i}, \end{array}$$ (13)
  where $X_{ant_{i}}^{t}$ is the updated ant number *i* position at iteration *t*, *a*_*i*_ is the minimum of random walk $X_{i}^{t}$ in *i* − *th* dimension, and *b*_*i*_ is the maximum of random walk $X_{i}^{t}$ in *i* − *th* dimension, *X*_*i*_ is defined in Eq 14, *c* and *d* are the lower and upper bounds of the random walk.
  $$\begin{array}{c} X\left(t\right)=[0,cumsum\left(2r\left(t_{1}\right)-1\right);cumsum\left(2r\left(t_{2}\right)-1\right);\\ ...;cumsum(2r\left(t_{T}\right)-1)], \end{array}$$ (14)
  where *cumsum* calculates the cumulative sum, and *r*(*t*) is a stochastic function characterized as in the Eq (15):
  $$\begin{array}{c} r\left(t\right)=\left\{\begin{array}{c} 1\;\text{if}\;rand>0.5\\ 0\;\text{if}\;rand\leq 0.5, \end{array}\right. \end{array}$$ (15)
  where *rand* is a random number created with uniform distribution in the interim of [0, 1].
  *c* and *d* parameters are adapted according to Eqs 16 and 17 to limit the range of the random walk around the given antlion.
  $$\begin{array}{c} c_{i}^{t}=\left\{\begin{array}{c} \frac{lb}{I}+X_{antlion_{j}}^{t}\;ifrand<0.5\\ \frac{lb}{I}+X_{antlion_{j}}^{t}\;otherwise, \end{array}\right. \end{array}$$ (16)
  $$\begin{array}{c} d_{i}^{t}=\left\{\begin{array}{c} \frac{ub}{I}+X_{antlion_{j}}^{t}\;ifrand>0.5\\ \frac{ub}{I}+X_{antlion_{j}}^{t}\;otherwise, \end{array}\right. \end{array}$$ (17)
  where *lb*, *ub* are the lower and upper limits for dimension *i*, *rand* is a random number drawn from uniform distribution and *I* is a factor that control the exploration/exploitation rate and is defined in Eq (18):
  $$\begin{array}{c} I=10^{w}\frac{t}{T}, \end{array}$$ (18)
  where *T* is the maximum number of iterations, *w* is a constant characterized taking into account the present iteration (*w* = 2 when *t* > 0.1*T*, *w* = 3 when *t* > 0.5*T*, *w* = 4 when *t* > 0.75*T*, *w* = 5 when *t* > 0.9*T*, and *w* = 6 when *t* > 0.95*T*). The constant *w* can modify the accuracy level of exploitation.
  Finally, the selection operation is applied where an antlion is replaced by an ant if the ant becomes fitter. As indicated by the above conditions ALO optimizer can be formulated as in the following algorithm 2.
  **Algorithm 2:** Antlion optimization (ALO) algorithm
  1: **Inputs**: *n* number of ants,
    *N* number of antlions,
    *T* maximum number of iterations.
  2: **Outputs**: The elitist antlion.
  3: Initialize a population of *n* ant’s positions and *N* antlion’s positions randomly.
  4: Calculate the fitness of all ants and antlions.
  5: Find the fittest antlion (the elite).
  6: *t* = 0.
  7: **while** *t* ≤ *T* **do**
  8:   **for all** *ant*_*i*_ **do**
  9:   Select an antlion utilizing the roulette wheel selection.
  10:   Slide ants towards the antlion; perform random pursuit around this selected antlion and normalize it.
  11:   Create a random walk for *ant*_*i*_ around the elite antlion and normalize it.
  12:   Update *ant*_*i*_ position as the average of the two obtained random walks.
  13:  **end for**
  14:  Calculate the fitness of all ants.
  15:  Replace an antlion with its corresponding ant it if gets to be fitter.
  16:  Update elite if an antlion gets to be fitter than the elite.
  17: **end while**
  18: Select the elitist antlion and its fitness.
3. **Moth-flame optimization (MFO**)
  Mirjalili proposed moth-flame optimization (MFO) in 2015 [26] that is motivated by the moths navigation approach. Moths depend on *transverse orientation* for navigation where a moth flies by keeping up a settled point concerning the moon. At the point when moths see the human-made artificial light, they endeavor to have a similar angle of the light to fly in the straight line. Moths and flames are the primary components of the artificial MFO algorithm. MFO is a population-based algorithm with the set of *n* moths are used as search operators. Flames are the best *N* positions of moths that are acquired so far. In this manner, every moth seeks around a flame and updates it if there should be an occurrence of finding a better solution. Given logarithmic spiral, a moth updates its position concerning a given flame as in the Eq (19) [26]:
  $$\begin{array}{c} S\left(M_{i},F_{j}\right)=D_{i}.e^{bt}.cos\left(2\pi t\right)+F_{j}, \end{array}$$ (19)
  where *D*_*i*_ shows the Euclidian distance of the *i* − *th* moth for the *j* − *th* flame, *b* is a constant to define the shape of the logarithmic spiral, *M*_*i*_ demonstrate the *i* − *th* moth, *F*_*j*_ shows the *j* − *th* flame, and *t* is a random number in [−1, 1].
  As might be found in the above equation, the next position of a moth is characterized with respect to a flame. The *t* parameter in the spiral equation describes how much the next position of the moth ought to be near the flame. The spiral equation permits a moth to fly around a flame and not necessarily in the space between flames taking into account both exploration and exploitation of solutions. With a specific end goal to further emphasize exploitation, we suppose that *t* is a random number in [*r*, 1] where *r* is linearly decreased from −1 to −2 through the span of emphasis and is called convergence constant. With this technique, moths tend to exploit their corresponding flames more precisely corresponding to the number of iterations. In order to upgrade the likelihood of converging to a global solution, a given moth is obliged to update its position utilizing one of the flames (the corresponding flame). In every iteration and in the wake of updating the flame-list, the flames are sorted based on their fitness values. After that, the moths update their positions as for their corresponding flames. To permit much exploitation of the best encouraging solutions, the number of flames to be taken after is diminished as for the iteration number as given in the Eq (20):
  $$\begin{array}{c} N_{flames}=round\left(N-l*\frac{N-1}{T}\right), \end{array}$$ (20)
  where *l* is the present iteration number, *N* is the maximum number of flames, and *T* demonstrates the maximum number of iterations. The moth-flame optimization (MFO) is exhibited in algorithm 3.
  **Algorithm 3:** Moth-flame optimization (MFO) algorithm
  1: **Inputs**: *n* number of moths,
    *N* number of flames,
    *T* maximum number of iterations.
  2: **Outputs**: *Best* the optimal flame position,
    *f*(*Best*) the optimal flame fitness value.
  3: Initialize a population of *n* flames positions randomly in the search space.
  4: **while** Stopping criteria is not met **do**
  5:   Update the *N* number of flames as in Eq (20).
  6:   Compute the fitness value for each moth.
  7:   **if** first iteration **then**
  8:    Sort the moths from best to worst as per their fitness and place the result in the flame list.
  9:   **else**
  10:   Merge the population of past moths and flames.
  11:   Sort the merged population from best to worst.
  12:   Select the best *N* positions from the sorted merged population as the new flame list.
  13:  **end if**
  14:  Calculate the convergence constant *r*.
  15:  **for all** *Moth*_*i*_ with *i* ≤ *n* **do**
  16:   Compute *t* as *t* = (*r* − 1)*rand + 1.
  17:   **if** *i* ≤ *N* **then**
  18:    Update *Moth*_*i*_ position as indicated by *Flame*_*i*_ applying Eq (19).
  19:   **else**
  20:    Update *Moth*_*i*_ position as indicated by *Flame*_*N*_ using Eq (19).
  21:   **end if**
  22:  **end for**
  23: **end while**
  24: Select the optimal position and its fitness.
4. **Chaotic maps**
  Chaotic systems are deterministic systems that display unpredictable (or even random) conduct and reliance on the underlying conditions. Chaos is a standout amongst the most prominent phenomena that exist in nonlinear systems, whose activity is complex and like that of randomness [27]. Chaos theory concentrates on the conduct of systems that take after deterministic laws yet seem random and unpredictable, i.e., dynamical systems [16]. To be considered as chaotic, the dynamical system must fulfill the chaotic properties, for example, delicate to starting conditions, topologically blend, dense periodic orbits, ergodic, and stochastically intrinsic [28]. Chaotic components can experience all states in specific reaches as per their own particular consistency without redundancy [27]. Because of the ergodic and dynamic properties of chaos elements, chaos search is more fit for slope-climbing and getting away from local optima than the random search, and in this way, it has been applied for optimization [27]. It is generally perceived that chaos is a principal method of movement basic all common phenomena. A chaotic map is a guide that shows some sort of chaotic behavior [28]. The basic chaotic maps in the literature are:
  - **Sinusoidal map:** represented by the Eq (21) [27]:
    $$\begin{array}{c} x_{k+1}=a\;x_{k}^{2}\;sin\left(\pi x_{k}\right), \end{array}$$ (21)
    which creates chaotic numbers in the range (0, 1) with *a* = 2.3.
  - **Singer map:** given in the Eq (22) [29]:
    $$\begin{array}{c} x_{k+1}=\mu \;\left(7.86x_{k}-23.31x_{k}^{2}+28.75x_{k}^{3}-13.3x_{k}^{4}\right), \end{array}$$ (22)
    with *x*_*k*_ ∈ (0,1) under the condition that *x*_0_ ∈ (0, 1), *μ* ∈ [0.9, 1.08].

**Table 1:** The leadership hierarchy of the grey wolves pack.

Agent	Responsibility
Alphas	Decision making for the whole pack regarding hunting, sleeping,…etc.
Beta	Help the alpha in decision-making and candidate to be the alpha.
Delta	This group have many rules and classes such as: Scouts: Responsible for watching the boundaries of the territory and warning the pack in case of any danger. Sentinels protect and guarantee the safety of the pack. Elders are the experienced wolves who used to be alpha or beta. Hunters help the alphas and betas when hunting prey and providing food for the pack. Caretakers are responsible for caring for the weak, ill, and wounded wolves in the pack.
Omega	Babysitters in the pack and it is the last wolf allowed eating.

### 2.2 The proposed chaos-based exploration rate

As specified in [30], meta-heuristic optimization strategies dependably utilize high-level data to guide its trial and error mechanism for discovering satisfactory solutions. All meta-heuristic algorithms apply particular tradeoff of randomization and local search. Two noteworthy parts control the performance of a meta-heuristic algorithm to be specific *exploitation* (intensification) and *exploration* (diversification) [30]. Diversification intends to produce various solutions in order to investigate the search space on the large scale while intensification intends to concentrate on the pursuit in a local region by exploiting the data that an immediate decent solution is found in this area. The good combination of these two components will guarantee that the global optimality is achievable [30].

**Fig 1:**
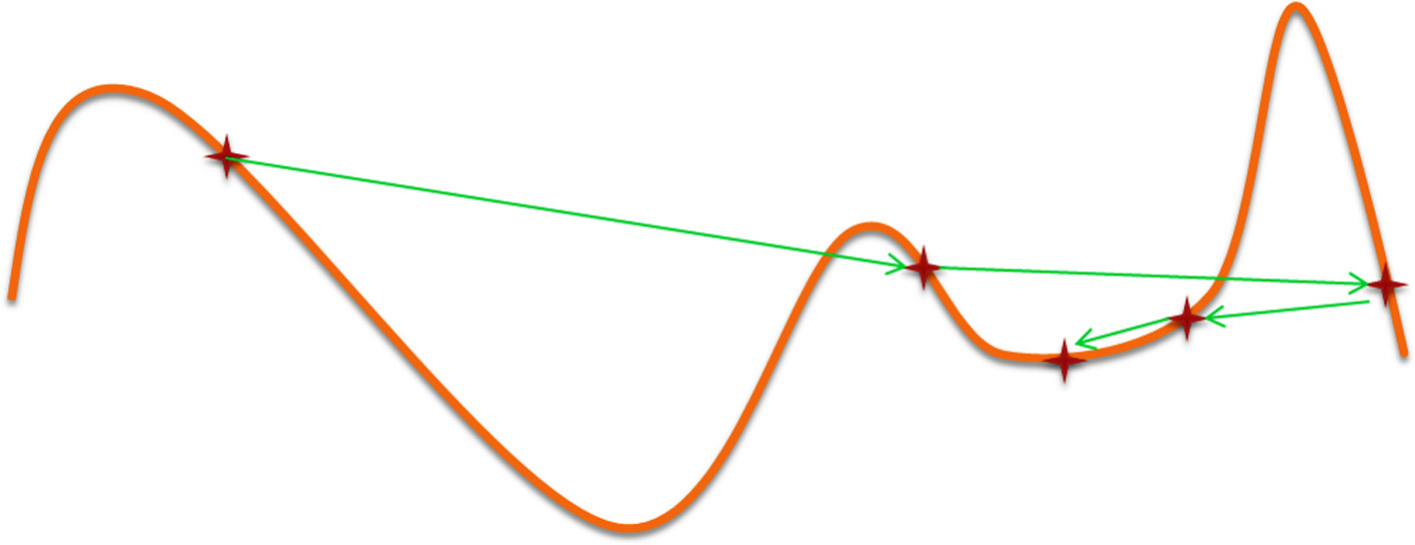
An example of leaving the promising region for the less promising one in 1-D case.

Some optimizers switches between exploration and exploitation gave some randomly generated values, for example, bat algorithm (BA) [31] and flower pollination algorithm (FPA) [32]. Numerous late investigates done in the optimization area to use a single versatile equation to accomplish both diversification and intensification with various rates as in GWO [23], ALO [25], and MFO [26]. In the both cases, the optimizer begins with large exploration rate and iteratively decrements this rate to improve the exploitation rate. The exploration rate takes after linear shape in GWO [23] and MFO [26] while it keeps the piece-wise linear shape in ALO [25]. In spite of the fact that exploration rate functions demonstrated proficient for tackling various optimization problems, despite this methodology has the following disadvantages:

1. **Stagnation:** once the optimizer approaches the end of the optimization process, it gets to be hard to get away from the local optima and discover better solutions since its investigation ability is turned out to be exceptionally constrained. This causes the algorithm to keep enhancing the solutions that have as of now been found, regardless of the possibility that they are local optima.
2. **Sub-optimal selection:** toward the start of the optimization prepare, the optimizer has extremely exploration ability yet with this enhanced explorative force it might leave a promising locale to less encouraging because of the oscillation and extensive training rates; see Fig 1 for such case in 1D representation.

These issues motivate using interleaved successive periods of exploration and exploitation all through the optimization time. In this way, when achieving a solution, the exploitation will be applied, followed by another exploration that permits the optimizer to jump to another promising region, then exploitation again to improve further the solution found. Chaotic systems with their intriguing properties, for example, topologically blend, ergodicity, and intrinsic stochasticity, which can be applied to adjust the exploration rate parameter, taking into account the required equilibrium between the diversification and intensification [16].

**Fig 2:**
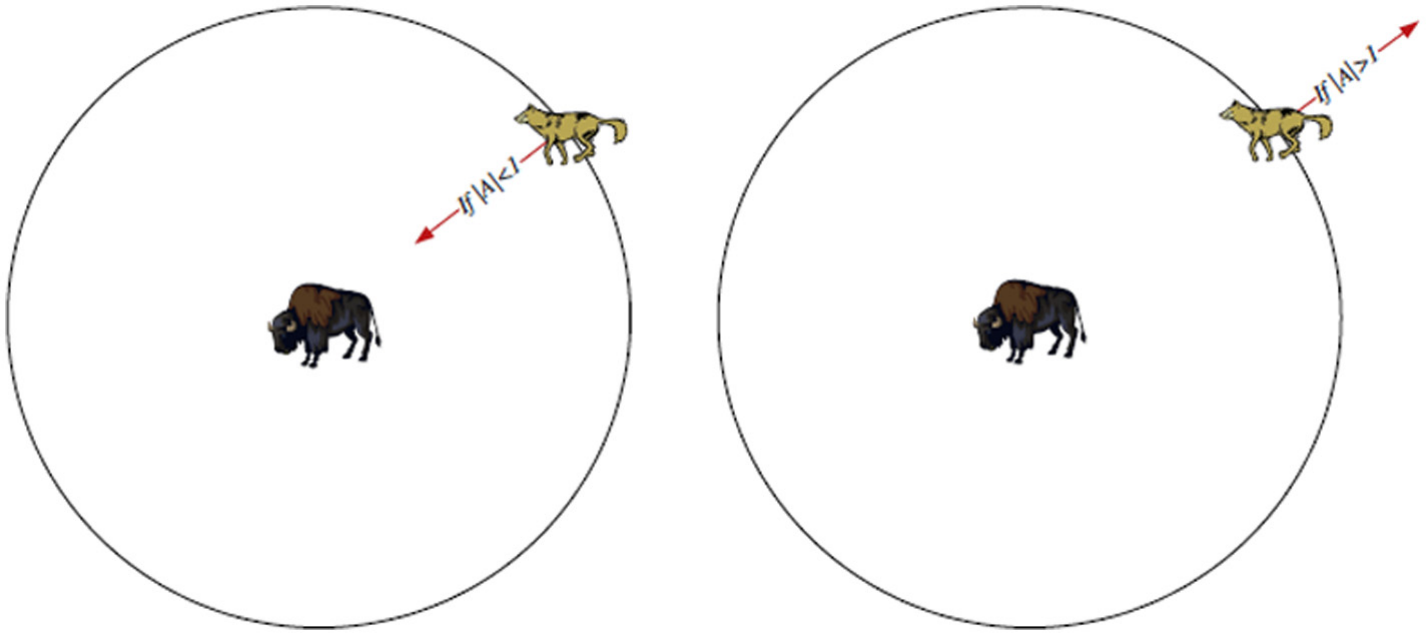
Exploration versus exploitation periods depending on the parameter *a* in GWO [23].

The diversification rate in the native GWO is called *a* and is linearly diminished from 2 to 0 throughout the course of iterations; as in the Eq (4). The *a* turns out to be under 1 to demonstrate the case that the wolf quit moving and approaches the prey. Along these lines, the following position of a wolf might be at any point between the wolf position and the prey position; see Fig 2 [23]. At the point when the estimation of *a* is greater than 1, wolves separate from each other to hunt down prey and merge to assault prey. Additionally, for the values greater than 1, the grey wolves are compelled to locate a fitter prey ideally; as in Fig 2 [23].

**Fig 3:**
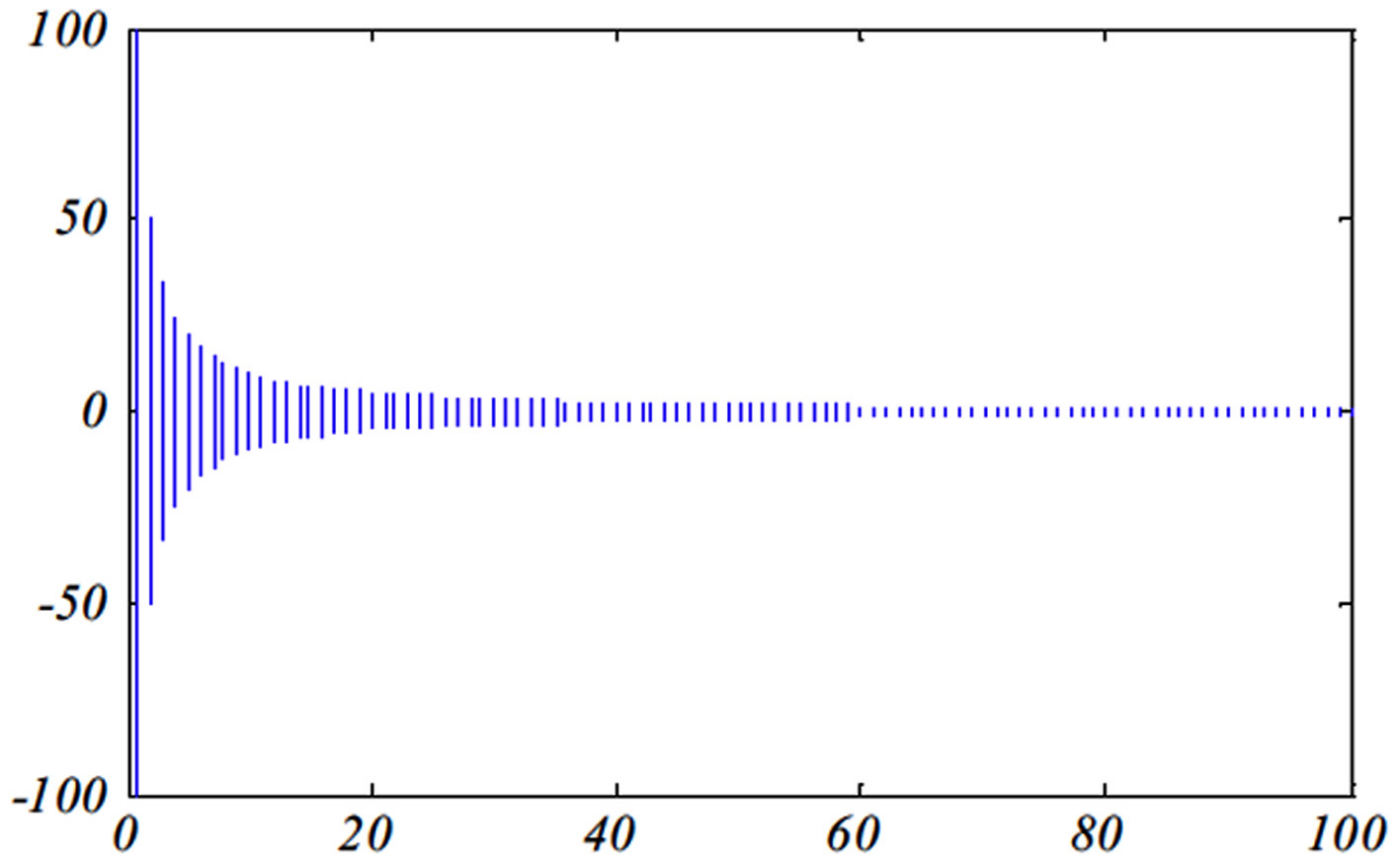
The shrinking of the random walk limits as per the parameter *I* [25].

In ALO, the exploration or exploitation are controlled by contracting the span of updating ant’s positions and imitates the ant sliding procedure inside the pits by using the parameter *I* as appeared in Fig 3. The span of ants’ random walks hypersphere is decreased adaptively, and this behavior can be mathematically modeling as in the Eq (23).

$$\begin{array}{c} I=10^{w}\frac{t}{T}, \end{array}$$ (23)

where *t* is the present iteration, *T* is the maximum number of iterations, *w* is a constant characterized taking into account the present iteration (*w* = 2 when *t* > 0.1*T*, *w* = 3 when *t* > 0.5*T*, *w* = 4 when *t* > 0.75*T*, *w* = 5 when *t* > 0.9*T*, and *w* = 6 when *t* > 0.95*T*). The constant *w* can conform the exactness level of exploitation.

**Fig 4:**
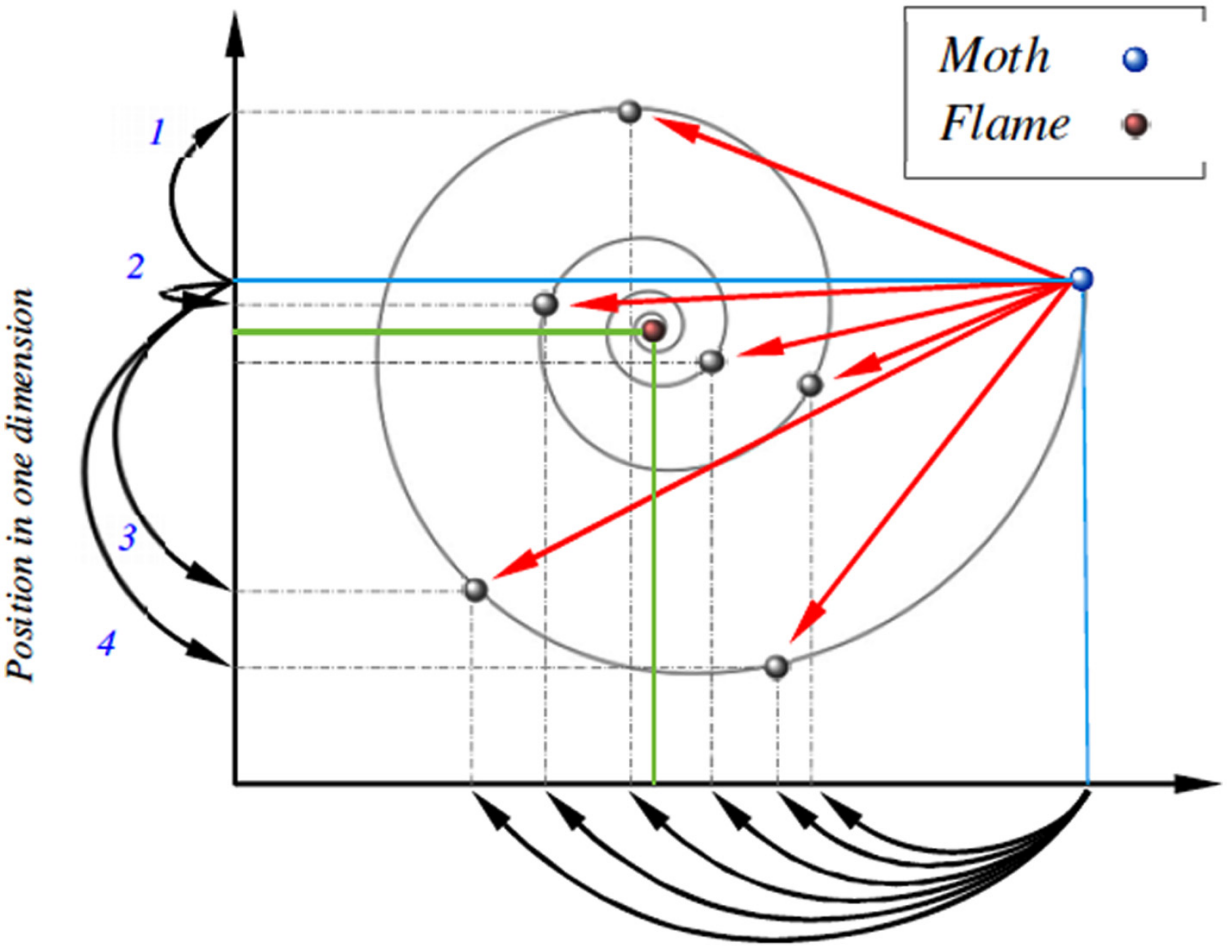
The possible positions of a given moth with respect to the corresponding flame [26].

In the case of MFO, the *T* parameter in the spiral equation characterizes how much the next position of the moth ought to be near (*the corresponding*) flame (*T* = −1 is the nearest position to the flame while *T* = 1 demonstrates the most remote one) as shown in Fig 4. The spiral equation permits a moth to fly around a flame and not as a matter of course in the space between them. In this manner, the exploration and exploitation of the search space can be ensured [26]. For switching between the exploration and exploitation operations, the parameter *a* is characterized and linearly decremented amid the optimization from -1 to -2 to control the furthest reaches of the *T* value. The definite moth position in a given dimension is computed as given in Eq (24).

$$\begin{array}{c} T=(a-1)*rand+1, \end{array}$$ (24)

where *rand* is a random number drawn from uniform distribution in the extent [0, 1] and *a* is computed as in the Eq (25).

$$\begin{array}{c} a=-1+t*\;frac-1Max_{Iter}, \end{array}$$ (25)

where *t* is the iteration number and *Max*_*Iter*_ is the total number of iteration.

**Fig 5:**
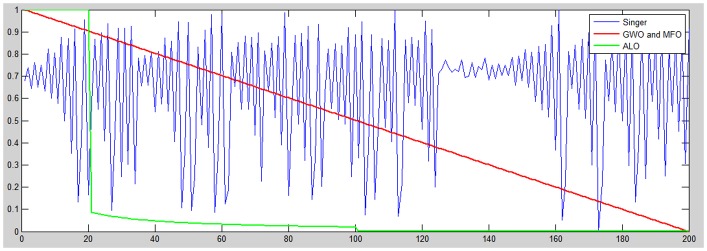
The Singer exploration rate versus the native exploration used as a part of GWO, ALO, and MFO.

**Fig 6:**
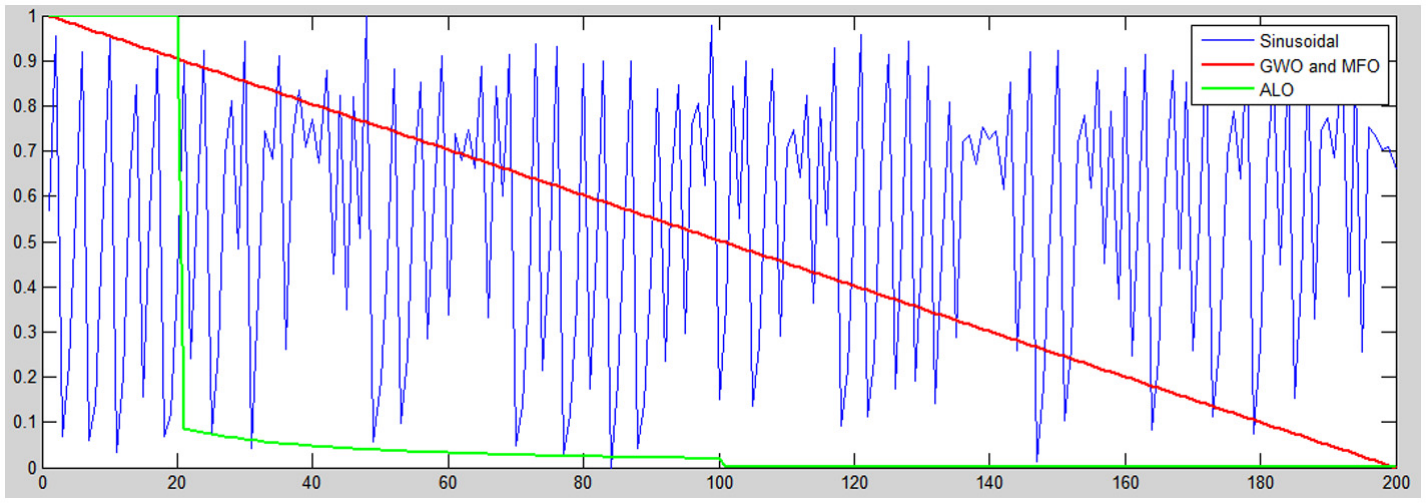
The Sinusoidal exploration rate versus the native exploration used as a part of GWO, ALO, and MFO.

In the proposed chaos adjustment of exploration rates, the adjustment of the parameter *a* in GWO is controlled by the given chaos function; see Fig 5. A comparative strategy is utilized to adjust the parameter *I* that confines the exploration rate in ALO; as in the Fig 6. While the same conduct is applied to MFO where the *a* parameter controls the parametric *t* value that decides the moth position regarding the corresponding flame; as shown in Fig 6. Two chaotic functions were used in this study to analyze their performance and impact on GWO, ALO, and MFO. The two chaotic functions have fit the progressive decrement of exploration and exploitation at successive periods are the *Singer* and *Sinusoidal* functions which are embraced here as examples.

### 2.3 Application of chaotic variants of GWO, ALO, and MFO for feature selection

In this section, the proposed chaotic variations are employed in feature selection for classification problems. For a feature vector sized *N*, the diverse feature subsets would be 2^*N*^ which is an immense space of features to be searched *exhaustively*. Therefore, GWO, ALO, and MFO are used adaptively to explore the search space for best feature subset. The best feature subset is the one with greatest *classification performance* and least *number of selected features*. The fitness function applied as a part of the optimizer to assess every grey wolf/antlion/moth position is as given by the following Eq (26):

$$\begin{array}{c} Fitness=\alpha \;\gamma_{R}\left(D\right)+\beta \;\frac{|C-R|}{|C|}, \end{array}$$ (26)

where *γ*_*R*_(*D*) is the classification performance of condition feature set *R* with respect to choice *D*, *R* is the length of selected feature subset, *C* is the aggregate number of features, *α* and *β* are two parameters relating to the significance of classification performance and subset length, *α* ∈ [0, 1] and *β* = 1 − *α*.

We can highlight that the fitness function maximizes the classification performance; *γ*_*R*_(*D*), and the proportion of the unselected features to the aggregate number of features; as in the term $\frac{|C-R|}{|C|}$. The above equation can be effectively changed over into a minimization by using the error rate as opposed to the classification performance and the selected features proportion as opposed to the unselected feature size. The minimization problem can be formulated as in the following Eq (27):

$$\begin{array}{c} Fitness=\alpha \;E_{R}\left(D\right)+\beta \;\frac{|R|}{|C|}, \end{array}$$ (27)

where *E*_*R*_(*D*) is the error rate for the classifier of condition feature set, *R* is the length of selected feature subset, and *C* is the aggregate number of features. *α* ∈ [0, 1] and *β* = 1 − *α* are constants to control the significance of classification performance and feature selection; *β* = 0.01 in the current experiments.

The principle characteristic of wrapper-based in feature selection is the usage of the classifier as a guide during the feature selection procedure. K-nearest neighbor (KNN) is a supervised learning technique that classifies the unknown examples and determined based on the minimum distance from the unknown samples to the training ones. In the proposed system, KNN is used as a classifier to guarantee the goodness of the selected features [33].

## 3 Results and Discussion

### 3.1 Data description

21 datasets in Table 2 from the UCI machine learning repository [34] are used as a part of the analyses and examinations results. The data sets were chosen to have different numbers of features and instances of the various problems that the proposed algorithms will be tried on.

**Table 2:** Description of the data sets used in the study.

Data set	No. features	No. instances
**Lymphography**	18	148
**WineEW**	13	178
**BreastEW**	30	569
**Breastcancer**	9	699
**Clean1**	166	476
**Clean2**	166	6598
**CongressEW**	16	435
**Exactly**	13	1000
**Exactly2**	13	1000
**HeartEW**	13	270
**IonosphereEW**	34	351
**KrvskpEW**	36	3196
**M-of-n**	13	1000
**PenglungEW**	325	73
**Semeion**	265	1593
**SonarEW**	60	208
**SpectEW**	22	267
**Tic-tac-toe**	9	958
**Vote**	16	300
**WaveformEW**	40	5000
**Zoo**	16	101

In this study, the wrapper-based approach for feature selection is using the KNN classifier. KNN is used in the experiments based on trial and error basis where the best decision of *K* is chosen (*K* = 5) as the best performing on all the data sets [33]. Through the training process, every wolf/antlion/moth position represents one solution (feature subset). The training set is used to assess the KNN on the validation set all through the optimization to guide the feature selection procedure. The test data are kept avoided the optimization and is let for final assessment. The global and optimizer-specific parameter setting is sketched out in Table 3. Every one of the parameters is set either as indicated by particular domain learning as the *α*, *β* parameters of the fitness function or based on trial and error on small simulations or regular in the literature.

**Table 3:** Parameter setting for experiments.

Parameter	Value(s)
No. of search agents	8
No. of iterations	100
Problem dimension	No. of features in the data
Search domain	[0 1]
No. Repetitions of runs	20
*α* parameter in the fitness function	[0.9, 0.99]
*β* parameter in the fitness function	1—[0.9, 0.99]
inertia factor of PSO	0.1
an individual-best acceleration factor of PSO	0.1

### 3.2 Evaluation criteria

Singular data sets are divided randomly into three different similar parts to be specific: training, validation, and testing sets in cross-validation manner. The apportioning of the data is repeated for 20 times to guarantee stability and statistical significance of the results. In every run, the following measures are recorded from the validation data:

- **Classification performance**: is a marker characterizes the classifier given the selected feature set and can be detailed as in the Eq (28).
  $$\begin{array}{c} Performance=\frac{1}{M}\sum_{j=1}^{M}\frac{1}{N}\sum_{i=1}^{N}Match\left(C_{i},L_{i}\right), \end{array}$$ (28)
  where *M* is the number of times to run the optimization algorithm to choose the feature subset, *N* is the number of samples in the test set, *C*_*i*_ is the classifier label for data sample *i*, *L*_*i*_ is the reference class label for data sample *i*, and *Match* is a function that yields 1 when the two input labels are the same and produces 0 when they are distinctive.
- **Mean fitness**: is the average of solutions obtained from running an optimization algorithm for various *M* running that can be given in Eq (29).
  $$\begin{array}{c} Mean=\frac{1}{M}\sum_{i=1}^{M}g_{*}^{i}, \end{array}$$ (29)
- **Standard deviation (std) fitness**: is a representation for the variety of the acquired best solutions found for running a stochastic optimizer for *M* different times. *Std* is employed as a pointer for the optimizer stability and robustness, though *std* is smaller this implies that the optimizer always converges to the same solution; while bigger values for *std* mean many random results as in the Eq (30).
  $$\begin{array}{c} Std=\sqrt{\frac{1}{M-1}\sum {\left(g_{*}^{i}-Mean\right)}^{2}}, \end{array}$$ (30)
  where *Mean* is the average characterized as in the Eq (29).
- **Selection features size**: demonstrates the mean size of the selected features to the aggregate number of features as in the Eq (31).
  $$\begin{array}{c} Selection=\frac{1}{M}\sum_{i=1}^{M}\frac{size(g_{*}^{i})}{D}, \end{array}$$ (31)
  where *size*(*x*) is the number of values for the vector *x*, and *D* is the number of selected features in data set.
- **Wilcoxon rank-sum test**: the null hypothesis is that the two examples originate from the same population, so any distinction in the two rank aggregates comes just from the inspecting error. The rank-sum test is described as the nonparametric version of the *t* test for two independent gatherings. It tests the null hypothesis that data in *x* and *y* vectors are samples from continuous distributions with equivalent medians against the option that they are not [35].
- **T-test**: is statistical importance demonstrates regardless of whether the contrast between two groups’ midpoints in all probability mirrors a “real” distinction in the population from which the groups were inspected [36].

The three embraced optimizers were evaluated against their corresponding chaos controlled one where the three optimizers; GWO, ALO, and MFO were employed with two chaotic functions (*Sinusoidal* and *Singer*) used as a part of the study. Also, the used optimizers and their chaotic variants are compared against one of the conventional optimizers namely particle swarm optimizer (PSO) [37].

### 3.3 Experimental results

**Fig 7:**
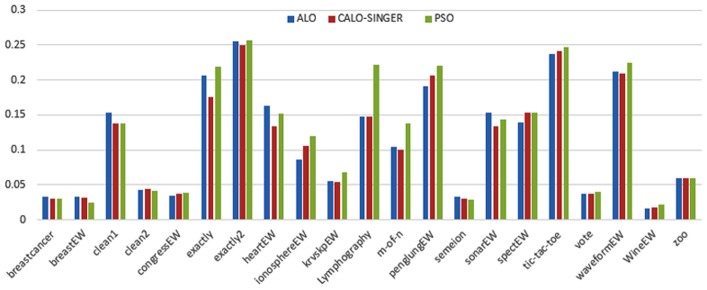
Mean fitness values for the ALO versus CALO using the Singer function.

**Fig 8:**
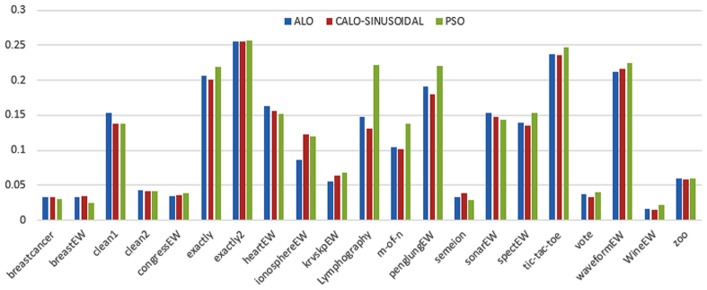
Mean fitness values for the ALO versus CALO using the Sinusoidal function.

**Fig 9:**
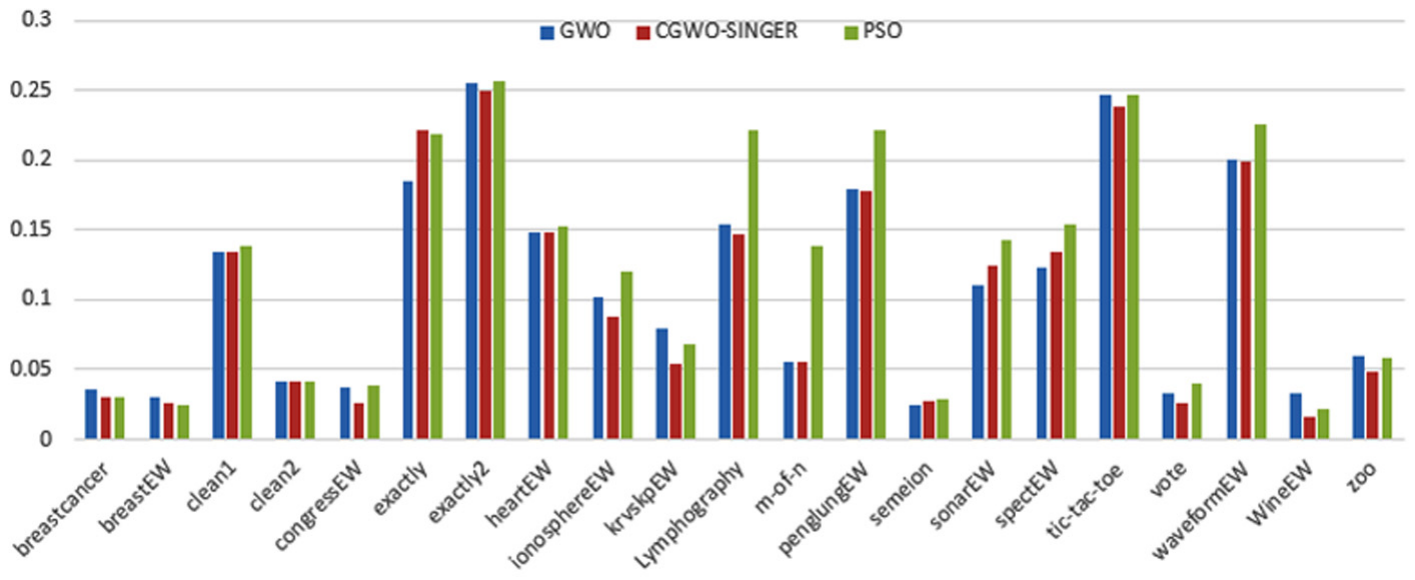
Mean fitness values for the GWO versus CGWO using the Singer function.

**Fig 10:**
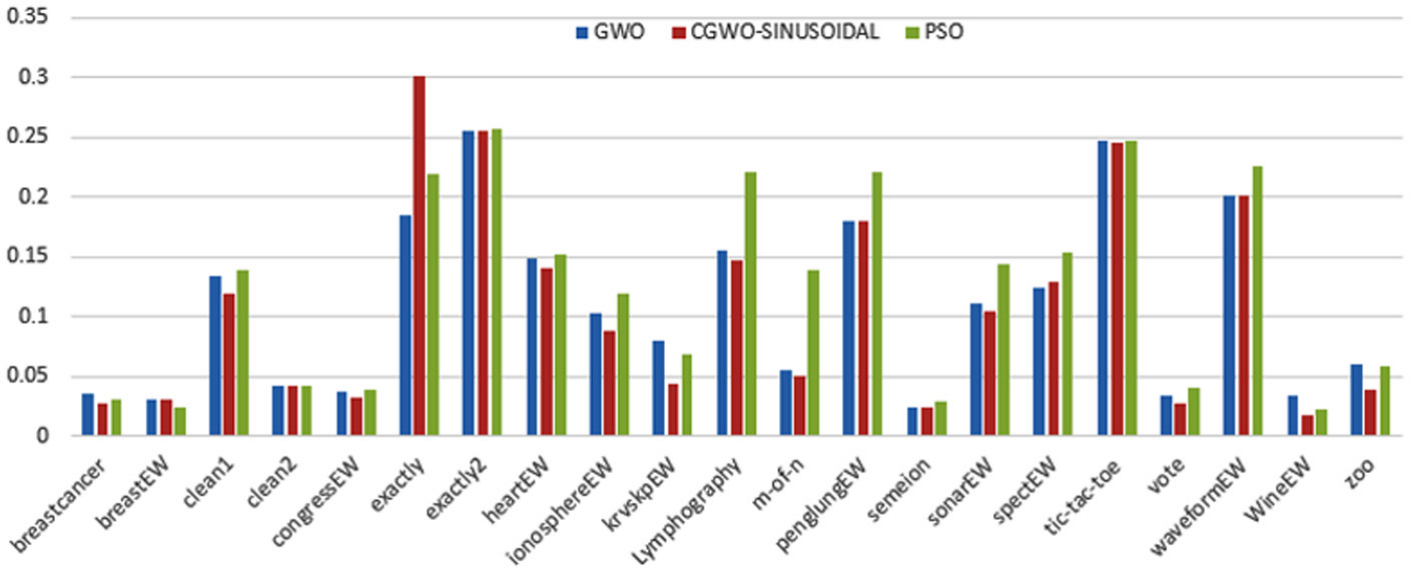
Mean fitness values for the GWO versus CGWO using the Sinusoidal function.

**Fig 11:**
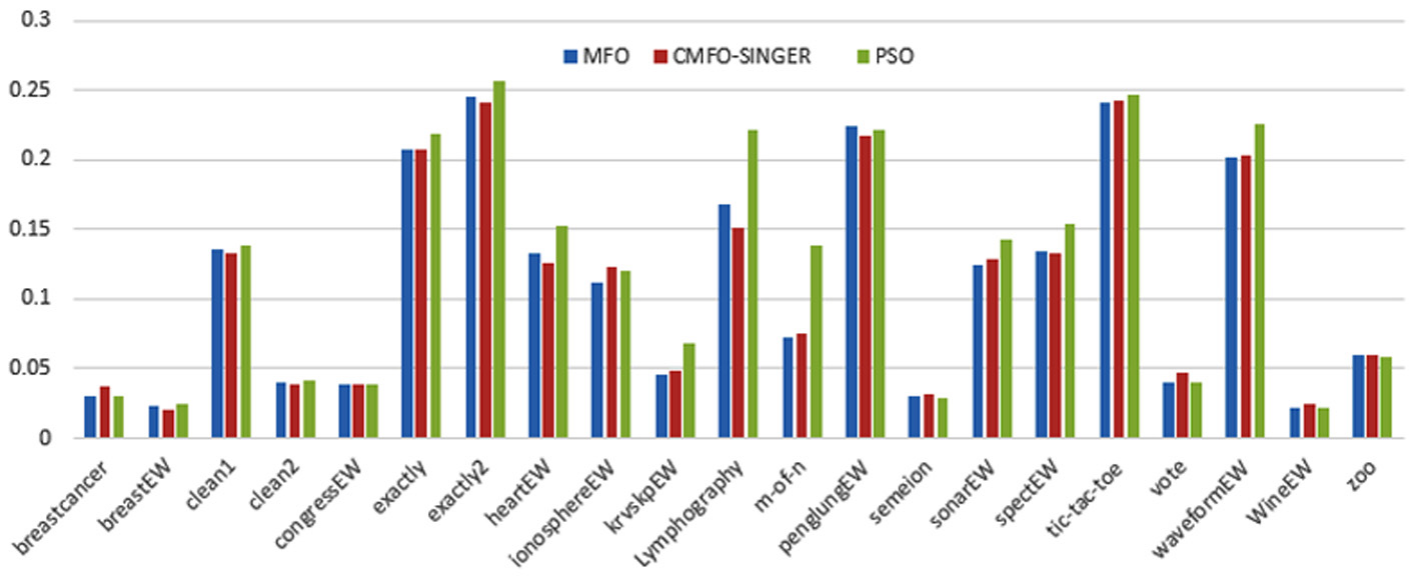
Mean fitness values for the MFO versus CMFO using the Singer function.

**Fig 12:**
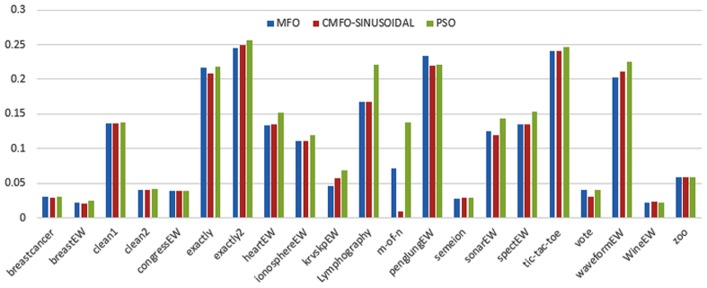
Mean fitness values for the MFO versus CMFO using the Sinusoidal function.

**Fig 13:**
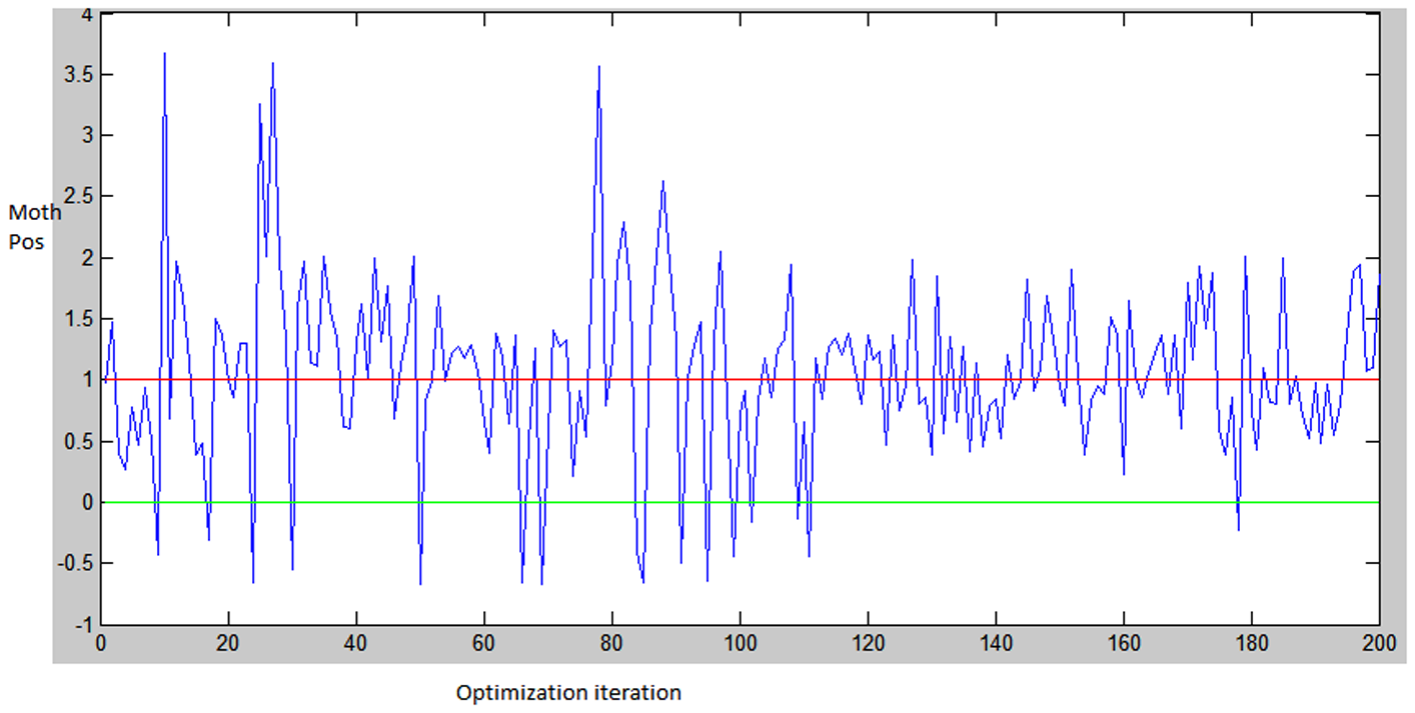
Possible positions for a moth using the different values for the a parameter.

Figs 7 and 8 outline the performance of the native ALO versus its chaotic one where the singer and the sinusoidal function is employed in all 21 data sets. We can observe from the figures that the chaotic ALO version [22] is superior in performance to the native ALO performance on most of the data set regardless of the used chaotic function. Comparable results are illustrated in Figs 9 and 10 applying the GWO. In the case of MFO, less enhance in performance is remarked where the chaotic and the native MFO have practically identical results as shown in Figs 11 and 12. The principle purpose behind this, a random value is utilized to choose the position of a moth that is joined with the used exploration rate, so it decreases its effect. Besides, the native MFO is relied upon to give variability in exploration toward the end stages of the optimization as found in Fig 13. Fig 13 demonstrates the conceivable positions for a moth in given dimension where the flame is situated at 1 while the moth is initially situated at 0 in that dimension at the different *a* values. We can highlight from the figure that it conceivable to provide exploration and exploitation at all the optimization stages and henceforth there is no improve in performance for the chaotic MFO.

**Fig 14:**
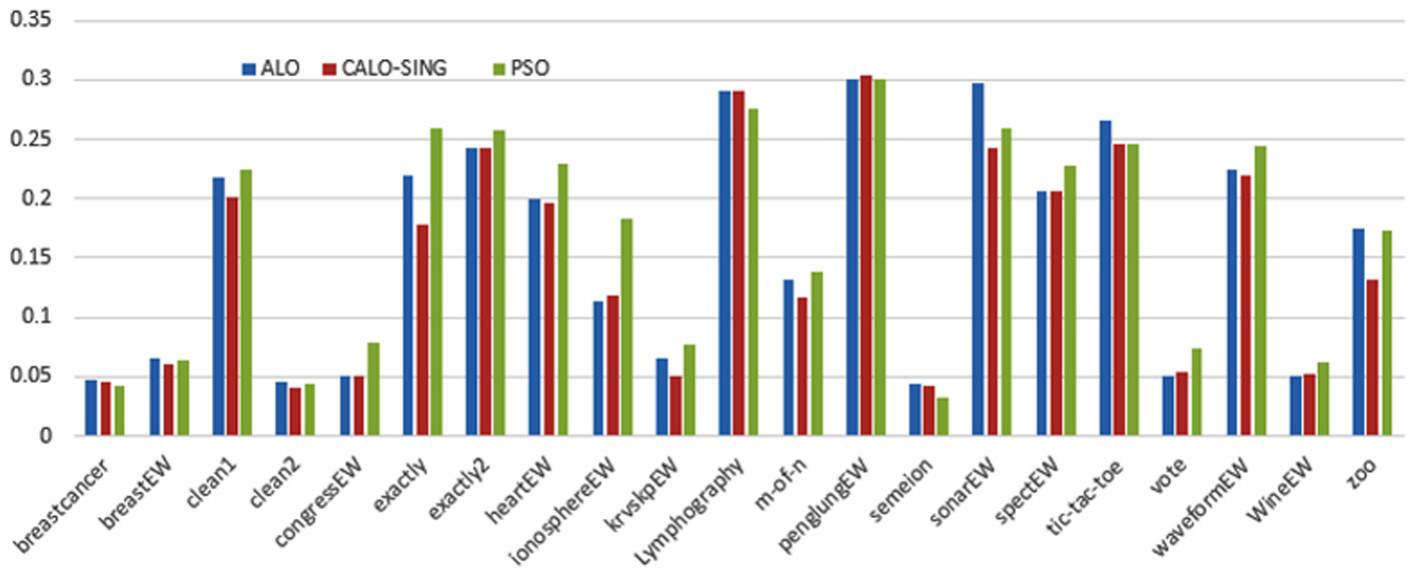
Average classification performance for the ALO versus CALO using the Singer function.

**Fig 15:**
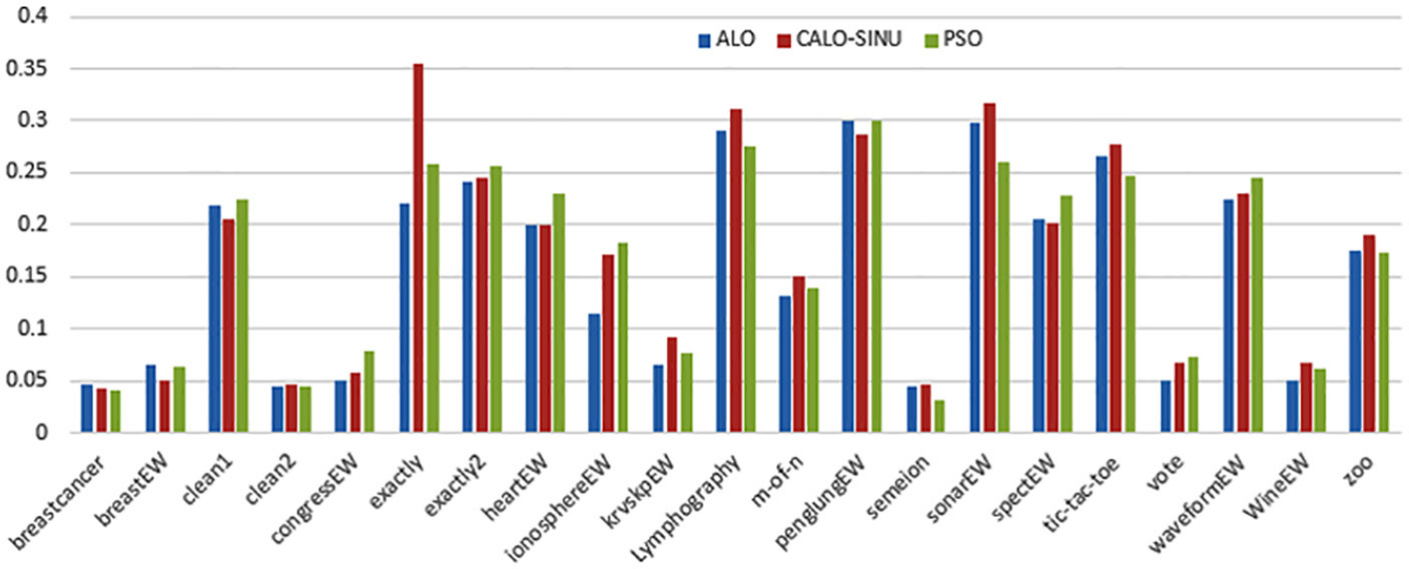
Average classification performance for the ALO versus CALO using the Sinusoidal function.

**Fig 16:**
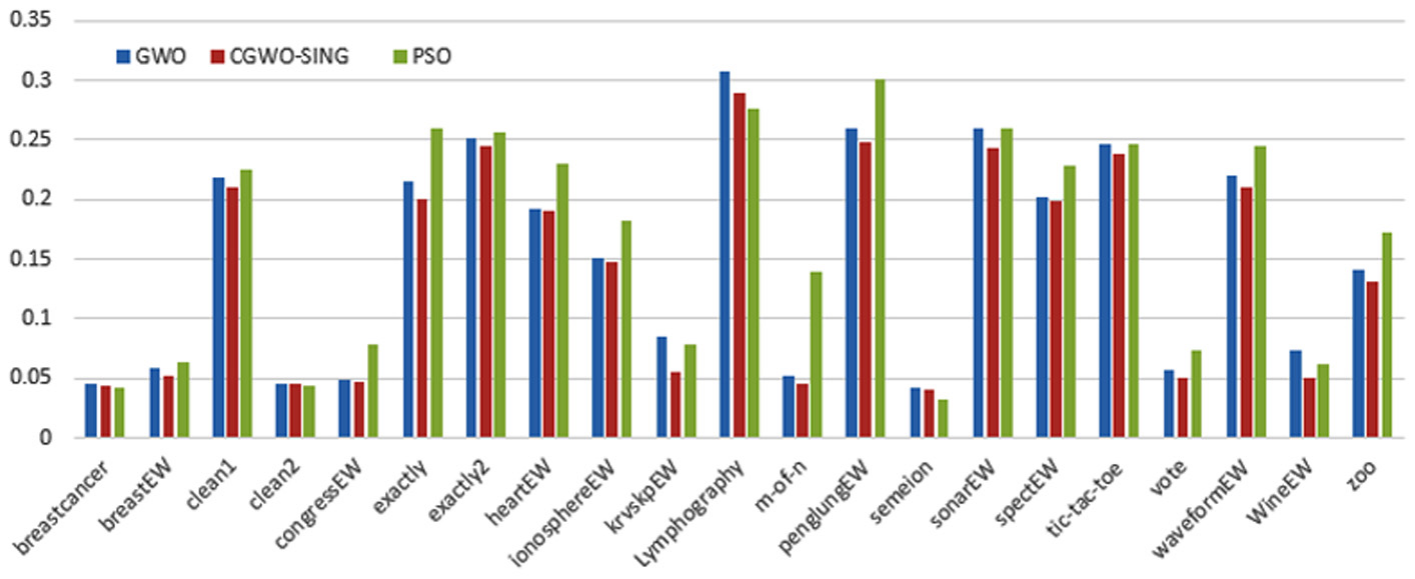
Average classification performance for the GWO versus CGWO using the Singer function.

**Fig 17:**
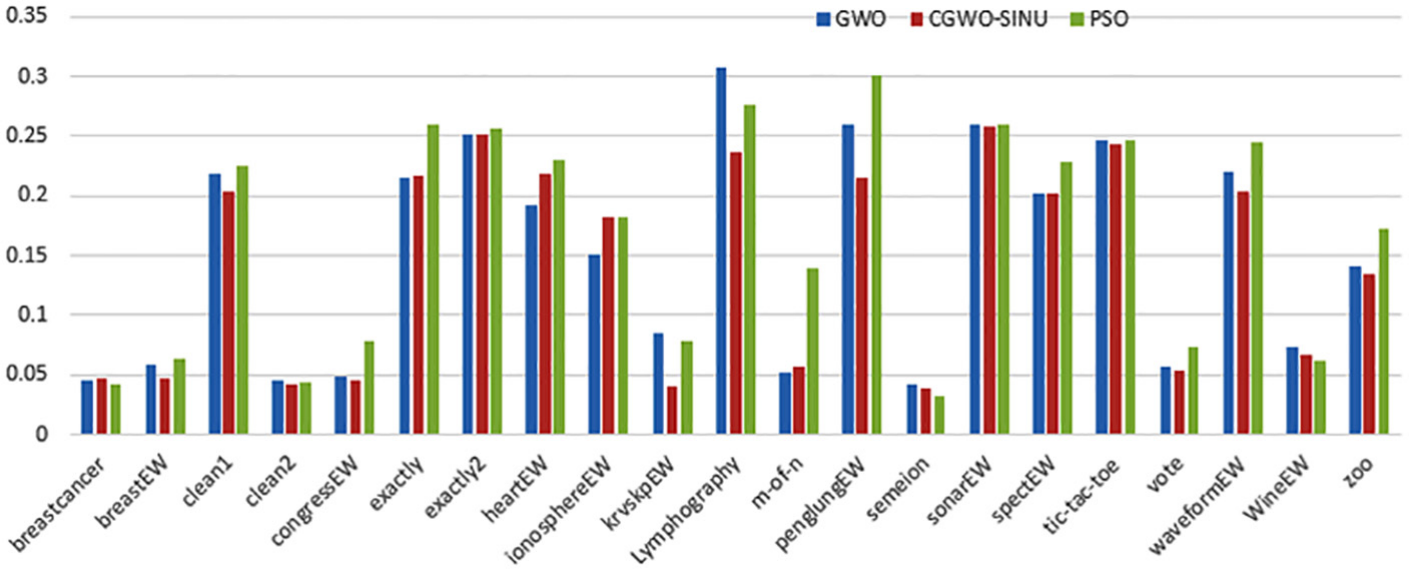
Average classification performance for the GWO versus CGWO using the Sinusoidal function.

**Fig 18:**
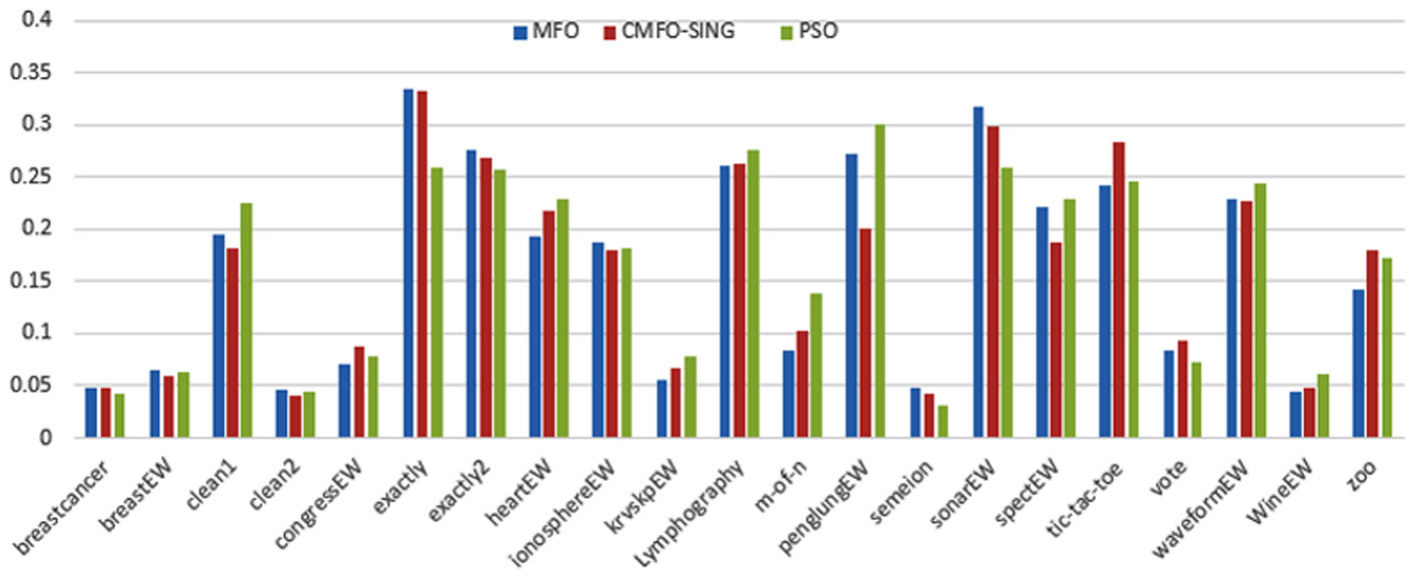
Average classification performance for the MFO versus CMFO using the Singer function.

**Fig 19:**
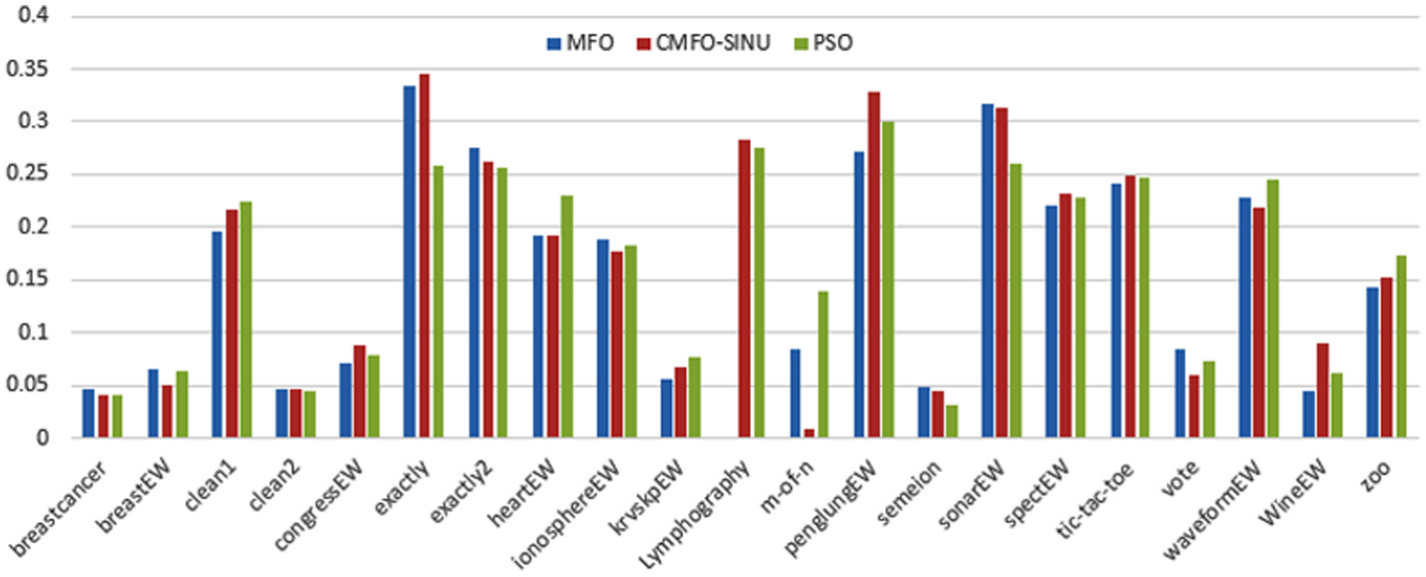
Average classification performance for the MFO versus CMFO using the Sinusoidal function.

**Fig 20:**
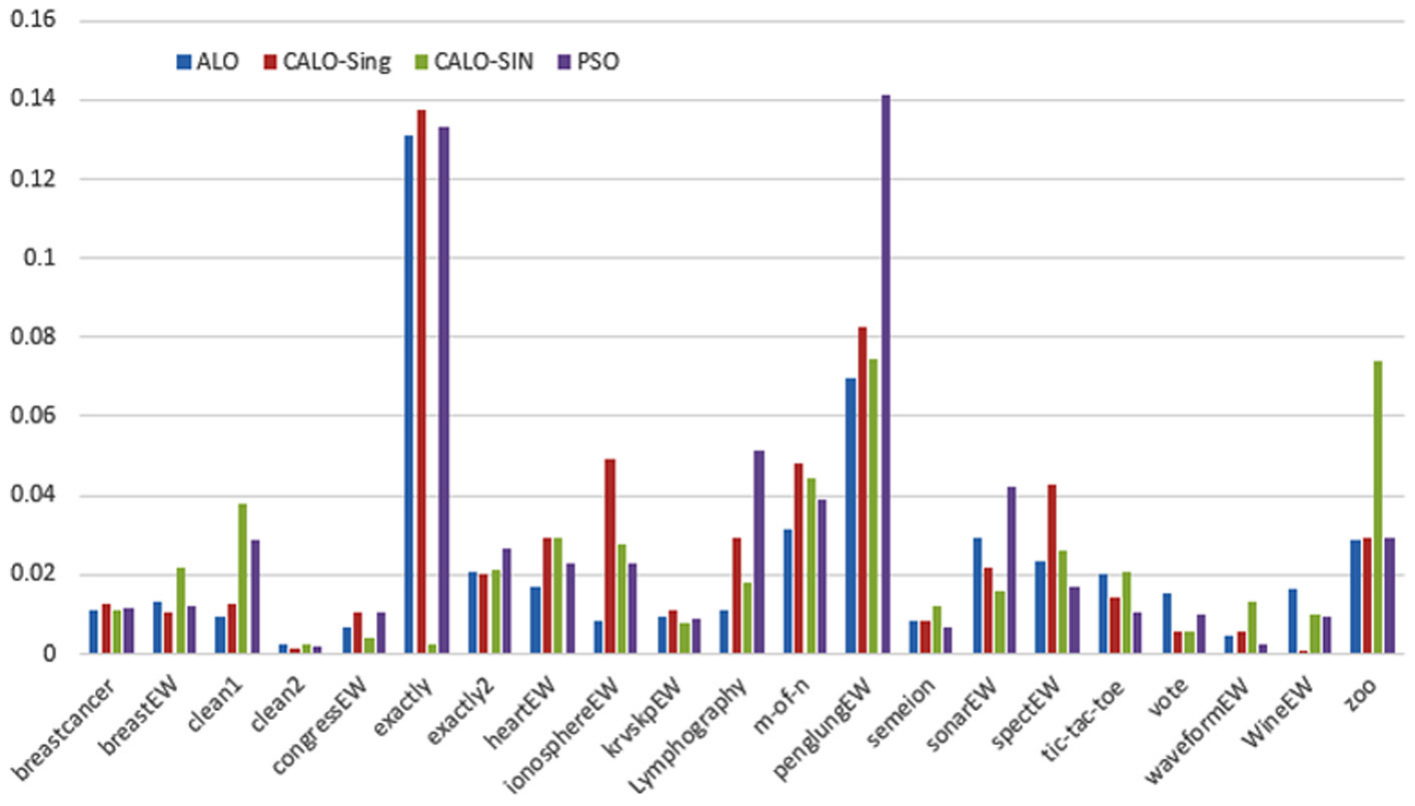
Standard deviation (std) of the obtained optimal fitness values for ALO versus CALO using the Singer and Sinusoidal functions.

**Fig 21:**
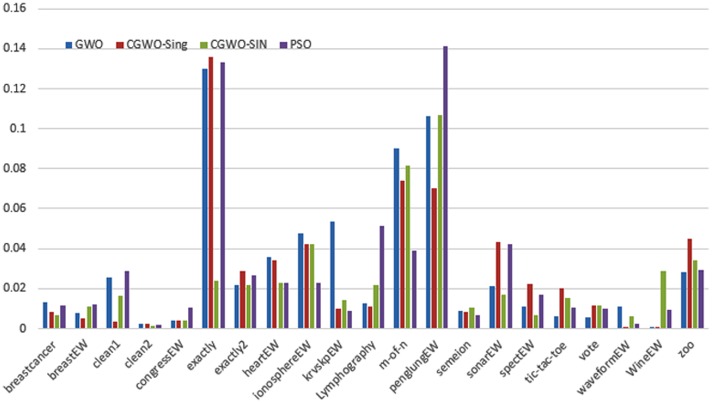
Standard deviation (std) of the obtained optimal fitness values for GWO versus CGWO using the Singer and Sinusoidal functions.

**Fig 22:**
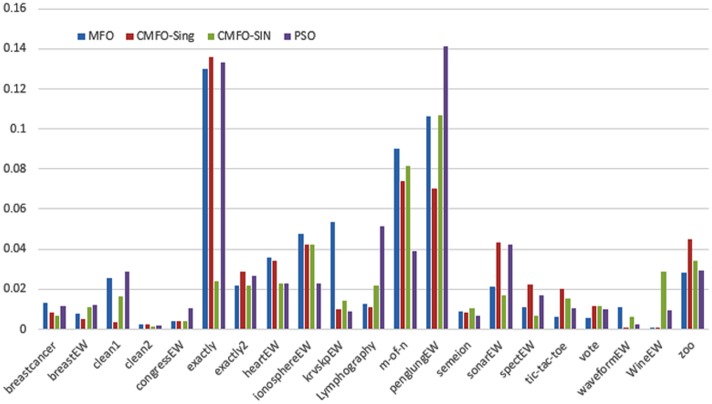
Standard deviation (std) of the obtained optimal fitness values for MFO versus CMFO using the Singer and Sinusoidal functions.

Comparative conclusions and comments can be found in Figs 14–19 that outline the performance of the selected features by the different optimizers on the test data. Moreover, we can remark that there is clear progress in performance for using chaotic versions with both ALO and GWO while the native MFO has practically identical performance on test data with it chaotic version. For assessing optimizer repeatability and capability to convergence to the global optimal regardless of the initial positioning of search agents, we have employed the standard deviation (std) of the acquired fitness function value over all the runs for the different optimizers. Figs 20–22 outline the obtained std values for the native and chaotic variants of three used optimizers. We can highlight from the figures that the chaotic versions of the optimizers have practically comparable std values in spite of the fact that they rely on upon chaotic functions as opposed to systematic functions which motivate using such models.

The secondary objective in the used fitness function is minimizing the number of selected features. Tables 4–6. We can remark that the three optimizers with their variants can optimize the number of selected features as well as improve the classifier performance. We can notice that this secondary objective is much achieved in the case of using the chaotic variants as the chaos allows the algorithms to continue exploring the space for the better solution even in the final periods of the optimization process. For assessing the significance of performance of the optimizers and their corresponding chaotic variants both the *t-test* and the *Wilixicon* test were measured in Table 7. Table 7 demonstrates the significant advance in utilizing the chaotic version of the ALO and GWO at a significance level of 0.05 while there is no remarkable progress in using the chaotic variant of MFO. A comparable conclusion can be derived from remarking the p-value of the t-test where the chaotic variant of ALO and GWO are much clear while there is no reasonable improve in performance for using the chaotic variant of MFO.

**Table 4:** Average selection features size for the chaotic and native ALO over all the data sets.

Data	CALO-SING	ALO	PSO	CALO-SIN
**Breastcancer**	**0.444**	0.519	0.519	0.481
**BreastEW**	0.500	0.478	**0.456**	0.522
**Clean1**	**0.329**	0.456	0.462	0.442
**Clean2**	**0.510**	0.679	0.518	0.661
**CongressEW**	**0.104**	0.167	0.375	0.200
**Exactly**	**0.564**	0.641	0.615	0.569
**Exactly2**	0.538	**0.077**	0.385	0.282
**HeartEW**	0.718	0.590	**0.538**	0.667
**IonosphereEW**	**0.069**	0.088	0.402	0.401
**KrvskpEW**	0.550	0.583	**0.426**	0.569
**Lymphography**	0.333	0.315	0.407	**0.204**
**M-of-n**	0.615	0.795	**0.590**	0.674
**PenglungEW**	**0.089**	0.190	0.425	0.317
**Semeion**	**0.377**	0.519	0.472	0.447
**SonarEW**	0.506	0.528	**0.439**	0.456
**SpectEW**	**0.121**	0.318	0.333	0.242
**Tic-tac-toe**	0.667	0.889	**0.556**	0.826
**Vote**	**0.104**	0.208	0.458	0.317
**WaveformEW**	0.733	0.883	**0.575**	0.767
**WineEW**	0.667	0.667	**0.590**	0.592
**Zoo**	0.375	0.479	**0.354**	0.417

**Table 5:** Average selection features size for the chaotic and native GWO over all the data sets.

Data	CGWO-SING	ALO	PSO	CGWO-SIN
**Breastcancer**	**0.444**	0.519	0.519	0.519
**BreastEW**	0.367	0.478	0.456	**0.267**
**Clean1**	**0.263**	0.456	0.462	0.315
**Clean2**	**0.259**	0.679	0.518	0.373
**CongressEW**	0.354	0.167	0.375	**0.125**
**Exactly**	**0.410**	0.641	0.615	**0.410**
**Exactly2**	0.205	**0.077**	0.385	0.154
**HeartEW**	**0.359**	0.590	0.538	0.385
**IonosphereEW**	0.255	**0.088**	0.402	0.304
**KrvskpEW**	**0.250**	0.583	0.426	0.370
**Lymphography**	0.241	0.315	0.407	**0.204**
**M-of-n**	**0.487**	0.795	0.590	**0.487**
**PenglungEW**	0.191	0.190	0.425	**0.160**
**Semeion**	**0.338**	0.519	0.472	0.341
**SonarEW**	**0.256**	0.528	0.439	0.261
**SpectEW**	**0.182**	0.318	0.333	0.212
**Tic-tac-toe**	0.593	0.889	**0.556**	**0.556**
**Vote**	0.208	0.208	0.458	**0.188**
**WaveformEW**	0.392	0.883	0.575	**0.375**
**WineEW**	**0.410**	0.667	0.590	**0.410**
**Zoo**	**0.271**	0.479	0.354	0.313

**Table 6:** Average selection features size for the chaotic and native MFO over all the data sets.

Data	CMFO-SING	ALO	PSO	CMFO-SIN
**Breastcancer**	**0.407**	0.519	0.519	0.481
**BreastEW**	0.489	0.478	**0.456**	0.467
**Clean1**	0.500	**0.456**	0.462	0.490
**Clean2**	0.528	0.679	0.518	**0.514**
**CongressEW**	0.542	**0.167**	0.375	0.479
**Exactly**	0.718	0.641	0.615	**0.513**
**Exactly2**	0.513	**0.077**	0.385	0.385
**HeartEW**	0.615	0.590	0.538	**0.513**
**IonosphereEW**	0.412	**0.088**	0.402	0.471
**KrvskpEW**	0.435	0.583	**0.426**	0.472
**Lymphography**	0.389	**0.315**	0.407	0.407
**M-of-n**	0.487	0.795	0.590	**0.462**
**PenglungEW**	0.456	**0.190**	0.425	0.416
**Semeion**	0.501	0.519	**0.472**	0.494
**SonarEW**	0.517	0.528	0.439	**0.422**
**SpectEW**	0.485	**0.318**	0.333	**0.318**
**Tic-tac-toe**	**0.556**	0.889	**0.556**	**0.556**
**Vote**	0.604	**0.208**	0.458	0.292
**WaveformEW**	**0.525**	0.883	0.575	0.575
**WineEW**	0.564	0.667	0.590	**0.436**
**Zoo**	0.458	0.479	**0.354**	0.479

**Table 7:** Significance tests for optimizers pairs.

Optimzer_1	Optimizer_2	Wilcoxon	T-test
**MFO**	**MFO-Sin.**	0.699	0.202
**MFO**	**MFO-Singer**	0.589	0.030
**MFO**	**PSO**	0.058	0.049
**MFO-Sin.**	**PSO**	0.05	0.048
**MFO-Singer**	**PSO**	0.047	0.049
**GWO**	**GWO-Sin.**	0.072	0.034
**GWO**	**GWO-Singer**	0.050	0.027
**GWO**	**PSO**	0.049	0.046
**GWO-Sin.**	**PSO**	0.043	0.048
**GWO-Singer**	**PSO**	0.037	0.039
**ALO**	**ALO-Sin.**	0.081	0.046
**ALO**	**ALO-Singer**	0.026	0.038
**ALO**	**PSO**	0.044	0.045
**ALO-Sin.**	**PSO**	0.048	0.044
**ALO-Singer**	**PSO**	0.047	0.049

**Fig 23:**
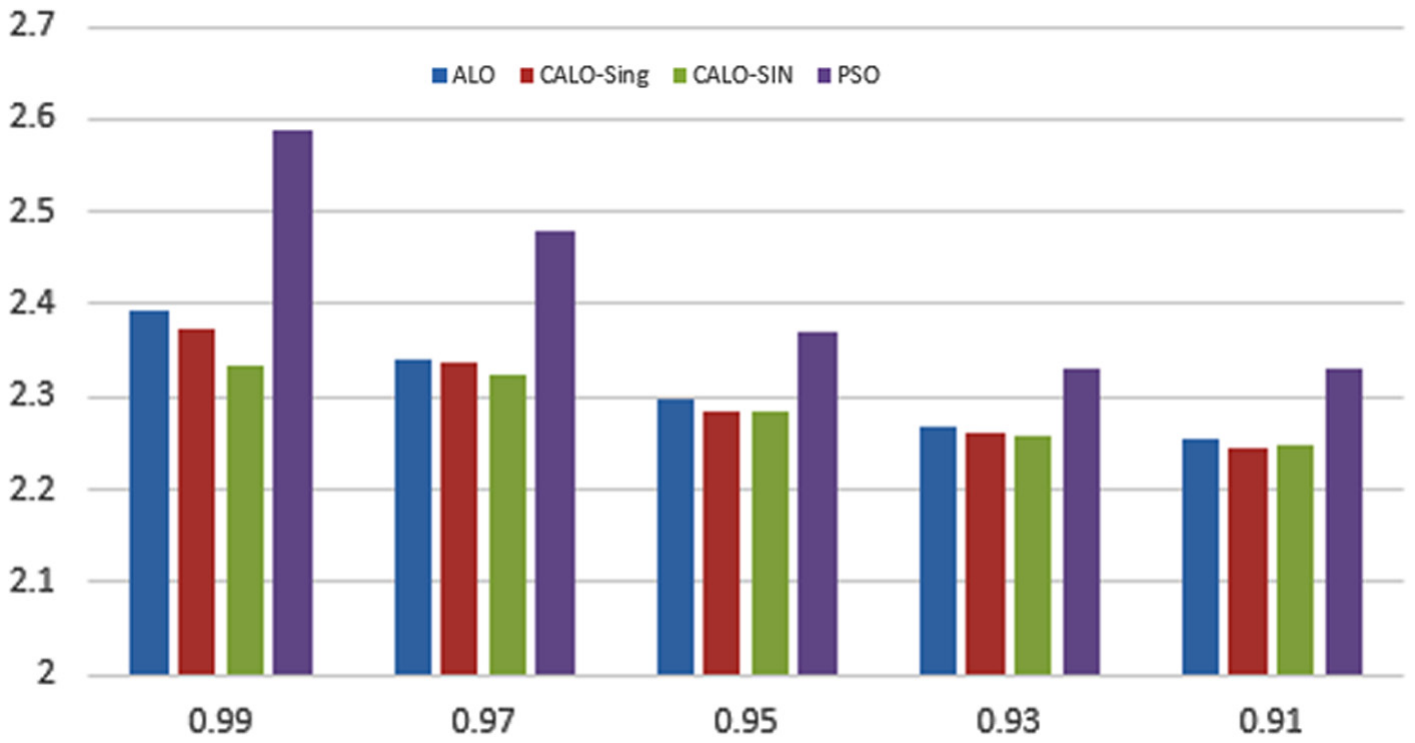
Fitness sum over all the used data sets for CALO versus ALO and PSO at a different setting of *α*.

**Fig 24:**
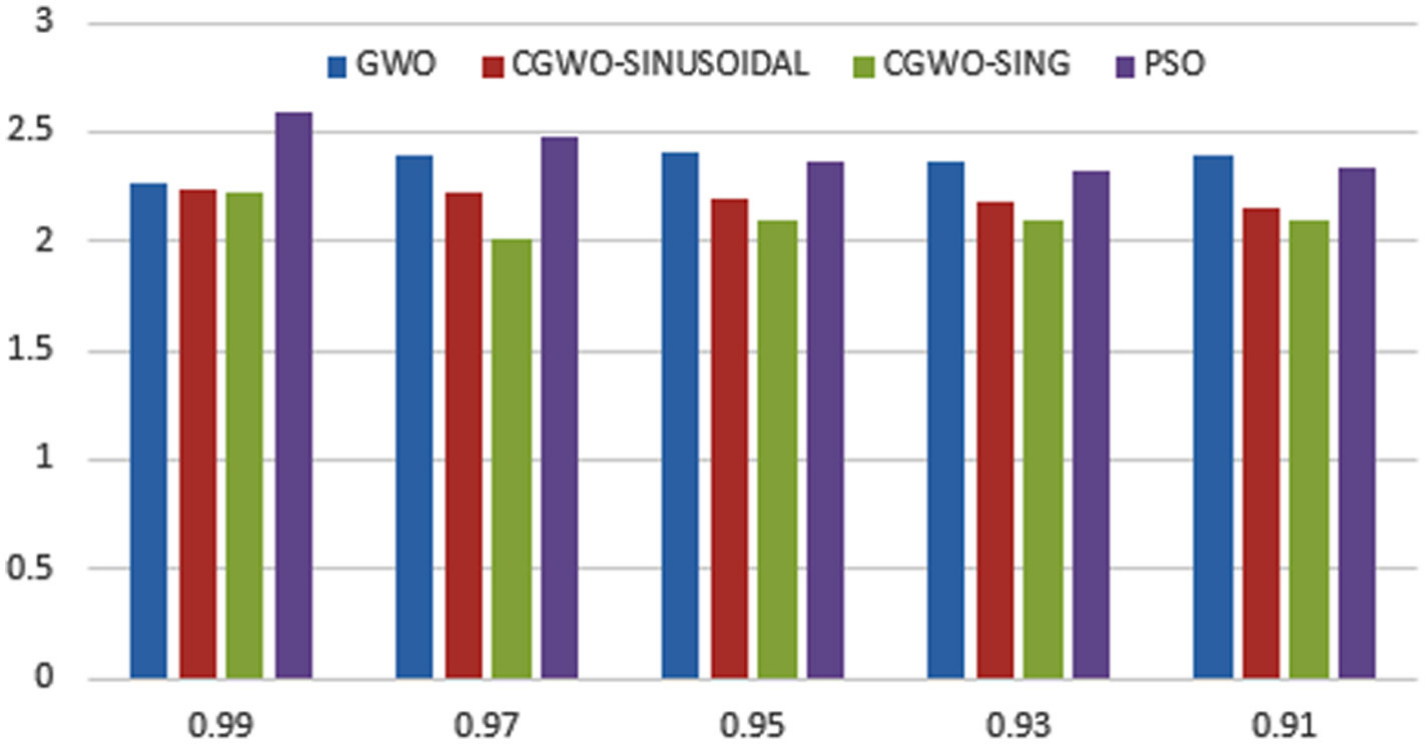
Fitness sum over all the used data sets for CGWO versus GWO and PSO at a different setting of *α*.

**Fig 25:**
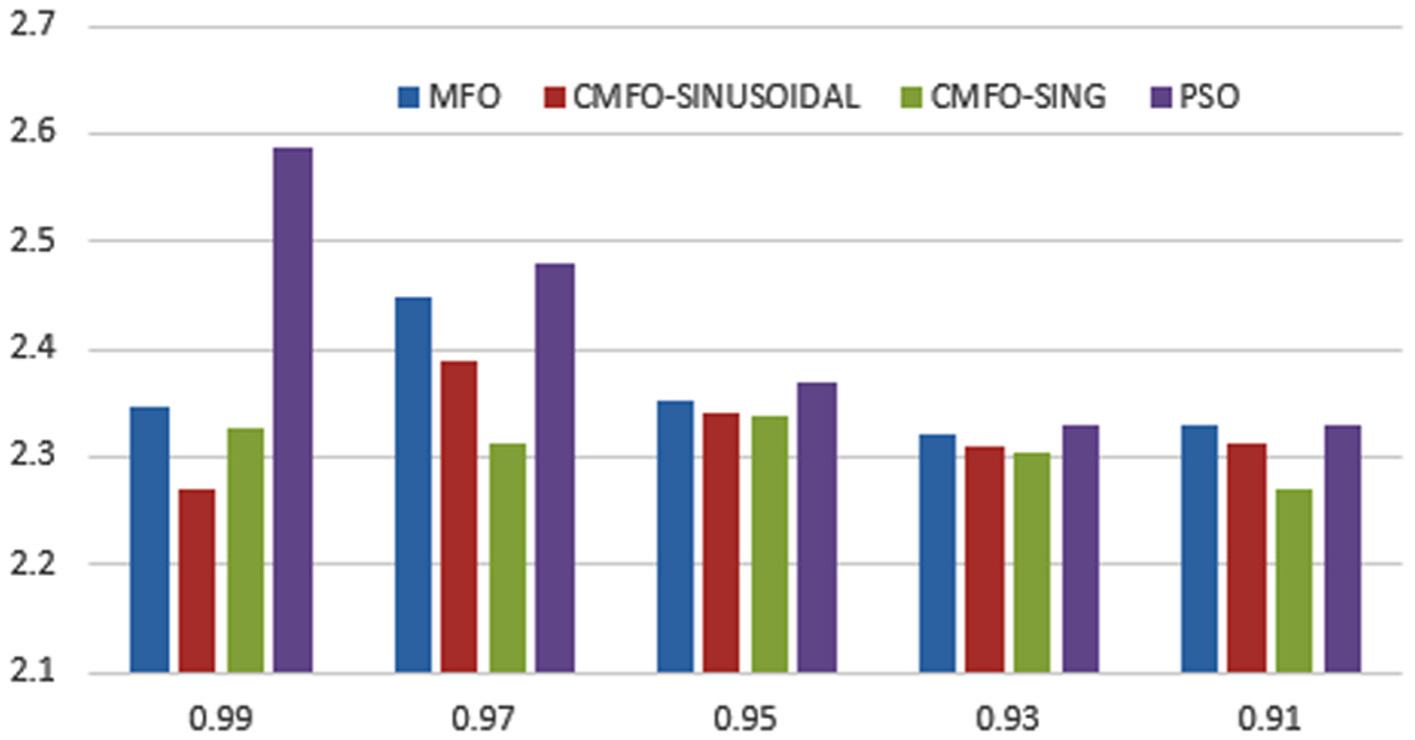
Fitness sum over all the used data sets for CMFO versus MFO and PSO at a different setting of *α*.

When using different weights of objectives *α* and *β* in the fitness function, we change the priority of each objective in the optimization process. Changing such weights affect the shape of the objective function and hence affects the performance of the used optimizers. The change in the shape of fitness function can result in appearance/disappearance of local minima and/or change of the global optima for such fitness function. Figs 23–25 depict the impact of changing the weight factor *α* on the performance of individual optimizers. We set the range of change of the *α* parameter to the range [0.99, 0.9] and the *β* parameter is set as 1 − *α* as the rational setting. The figures display the obtained sum of fitness function values obtained by the 10 different employed optimizers over all the data sets used. We can view from the figures that the chaotic versions of the optimizers still performs well with changing the shape of the fitness function which prove the capability of such variant to tolerate local optimal and convergence to a global one.

## 4 Conclusions

In this study, two chaos functions were employed to control the exploration rate for three of the modern bio-inspired optimization algorithms. The fundamental assumption of the work evaluates the impact of using such chaos functions as a replacement for the originally used linear or quasi-linear functions. The study evaluates the proposed two chaotic versions of the algorithms as well as the original ones in the domain of machine learning for feature selection. The results were conducted on a set of data sets drawn from the UCI repository and were assessed using different assessment indicators. The study found that utilizing exploration with high rate toward the end stages of optimization offers the algorithm some assistance with avoiding the local optima and premature convergence. Additionally, using the exploitation with high rate at the beginning of optimization helps the algorithm to select the most promising regions in the search space. Henceforth, the paper proposes using successive periods of exploration and exploitation during the optimization time. The chaos functions are the illustration of such functions that can provide successive exploration and exploitation and thus it provides excellent performance when used with GWO and ALO.
